# Improving polarized neutron reflectometry experiments on soft-matter samples: optimization of the solid substrate structure

**DOI:** 10.1107/S160057672600539X

**Published:** 2026-07-22

**Authors:** Ivan P. Yakimenko, Alessandra Luchini, Joshaniel F. K. Cooper, Sebastian Köhler, Tommy Nylander, Sean Langridge, Christy J. Kinane, Andrew J. Caruana, Björgvin Hjörvarsson, Max Wolff, Alexei Vorobiev, Sergiy Valyukh, Fredrik Eriksson, Jens Birch, Kenneth Järrendahl

**Affiliations:** a Thin Film Physics Division, Department of Physics, Chemistry and Biology (IFM), Linköping University, SE-581 83 Linköping, Sweden; b Department of Physics and Geology, University of Perugia, via Alessandro Pascoli, 06123 Perugia, Italy; c Department of Physics and Geology, CNR-IOM c/o University of Perugia, via Alessandro Pascoli, 06123 Perugia, Italy; d European Spallation Source ERIC Lund Sweden; e Division of Physical Chemistry, Department of Chemistry, Lund University, 221 00 Lund, Sweden; f LINXS Institute of Advanced Neutron and X-ray Science, 223 70 Lund, Sweden; g NanoLund, Lund University, Lund, Sweden; h School of Chemical Engineering and Translational Nanobioscience Research Center, Sungkyunkwan University, 16419 Suwon, Republic of Korea; i ISIS Neutron and Muon Source, Rutherford Appleton Laboratory, Chilton, Oxon OX11 0QX, United Kingdom; j Department of Physics and Astronomy, Material Physics, Uppsala University, SE-751 20 Uppsala, Sweden; k Institut Laue–Langevin, 71 Avenue des Martyrs, CS 20156, 38042 Grenoble Cedex 9, France; Oak Ridge National Laboratory, USA

**Keywords:** polarized neutron reflectometry, magnetic reference layers, solid supported lipid bilayers, Fisher information, experiment optimization, soft-matter samples

## Abstract

In this study we use Fisher information within the software *HOGBEN* to optimize the structure of magnetic reference layers to maximize the information gained from polarized neutron reflectometry (PNR) measurements on soft-matter and biological samples. Our approach is based on simulating the reflectivity from an experimentally determined dataset of a supported lipid bilayer. A set of reflectivity curves is calculated by varying parameters associated with the substrate assembly containing a magnetic reference layer. For each of the calculated reflectivity curves, we quantified the sensitivity of the simulated data to the sample structure. Our Fisher information analysis predicts that decreasing the thickness of the capping layer combined with increasing the magnetic scattering length density of the magnetic reference layer will reduce measurement time of similar PNR experiments by up to a factor of 5.6 or, equivalently, reduce parameter uncertainties at fixed beam time by the same factor relative to the reference experiment. Within a practically realizable SiO_2_ capping layer window of 200–400 Å, the predicted gain remains substantial (*G* ≈ 2.7). Because the parameters determining reflectivity curves are often correlated, such information is difficult to obtain in any other way. Experimental validation of these predictions on substrate assemblies synthesized according to the proposed designs is the natural next step. We established a theoretical method that can also be implemented within the context of other neutron reflectometry experiment. The approach allows the optimization of PNR experiments and will enable more straightforward and robust extraction of relevant parameters as well as more efficient use of neutron beam time.

## Abstract 2

We demonstrate a Fisher-information-based strategy for optimizing the solid substrate structure in polarized neutron reflectometry experiments on soft-matter samples. The approach identifies magnetic reference layer designs that improve experimentally accessible information and provides a general framework for more efficient reflectometry experiment planning.

## 1. Introduction

Neutron reflectometry (NR) is a technique to study the structure of buried interfaces and is particularly useful for hard and soft condensed matter thin films (Kirby *et al.*, 2012 ▸; Gerelli, 2020 ▸; Gerelli & Fragneto, 2025 ▸; Skoda, 2019 ▸; Wolff & Gutfreund, 2021 ▸; Wolff *et al.*, 2024 ▸), including supported lipid bilayers (SLBs), *i.e.* lipid bilayers deposited on the surface of a solid substrate assembly (Wacklin, 2010 ▸) (and hereafter referred to as ‘sample’). In soft-matter research, isotopic labelling, which exploits the different neutron scattering cross section associated with the hydrogen isotopes protium and deuterium, provides unique opportunities for NR (Lakey *et al.*, 2022 ▸). Therefore, selective replacement of hydrogen with deuterium is a powerful approach to enhance the neutron contrast of specific components within the sample. Deuterated solvents, such as D_2_O or D_2_O-based buffer, can likewise be used to tune the contrast, *i.e.* the scattering length density (SLD) difference between the soft-matter sample and the solvent, thereby improving sensitivity to interfacial structure and increasing the information content of the reflectivity data.

In general, NR provides information such as thickness, solvent penetration, composition or roughness of adsorbed layers at interfaces (Cousin & Fadda, 2020 ▸) by fitting a model to the experimental NR data. A common data-analysis approach is to describe the sample as a stack of layers each described by four parameters: (i) thickness, (ii) SLD, (iii) solvent volume fraction and (iv) surface roughness (Cousin & Chennevière, 2018 ▸). When fitting model parameters to a single experimental NR dataset it is often challenging to obtain a unique solution describing the experimental data, since just the reflected intensity |*r*(*Q*)|^2^ is measured and the phase of the complex reflection amplitude *r*(*Q*) is lost (the ‘phase problem’) (Majkrzak & Berk, 1998 ▸; Majkrzak *et al.*, 2003 ▸). This limitation is overcome by collecting different datasets corresponding to the same sample in contact with different solvents, which are analysed simultaneously with the same structural model (Heinrich *et al.*, 2020 ▸). This approach, known as solvent contrast variation, relies on the assumption that the sample structure is not affected by solvent rinsing steps, which are required *e.g.* to replace one solvent with another with different D_2_O content. This assumption might be less valid for weakly adsorbed thin films, where the solvent rinsing might actually remove part of the layer from the substrate or affect the layer structure in other ways, like increasing the hydration.

Another possibility to generate a variable contrast with the sample is to use a substrate assembly (SUB) with a magnetic reference layer (MRL). By applying an external magnetic field, the MRL, which is buried within or on top of the substrate, is magnetized in a specific direction and can therefore be probed with neutrons of different spin states. The MRL enables the collection of two independent datasets, which have different magnetic contrasts for the cases of the neutron spin being parallel and antiparallel to the applied magnetic field direction, and the possibility of decoupling the model parameters without exchanging the solvent (Zhang *et al.*, 1995 ▸; Majkrzak & Berk, 1998 ▸; Majkrzak *et al.*, 2003 ▸; Dabkowska *et al.*, 2017 ▸). Although solvent contrast variation is the first choice for investigating soft-matter thin films, the use of SUBs with MRLs has been shown to significantly increase the information gained from polarized NR (PNR) measurements of a range of soft-matter samples (Clifton *et al.*, 2015 ▸; Luchini *et al.*, 2025 ▸; Ayscough *et al.*, 2025 ▸; Holt *et al.*, 2009 ▸; Junghans *et al.*, 2015 ▸), including SLBs. In most of these studies the used SUBs were composed of a silicon crystal with a permalloy MRL and a gold capping layer. An alternative type of SUB that included an Fe MRL and a silicon oxide capping layer was introduced by Dikaia *et al.* (2024 ▸). The silicon oxide surface offers some advantages, as SLBs can be conveniently formed by vesicle fusion and the surface roughness is generally lower than that of the gold capping layer. Recently, the application of Fe MRLs and a silicon oxide capping layer was reported for the investigation of SLBs produced by spreading sponge-phase nanoparticles (L_3_ NPs) (Luchini *et al.*, 2025 ▸). Within the L_3_ NPs, the single acyl chain lipids GMO-50 and DGMO are organized as a continuous lipid bilayer (Valldeperas *et al.*, 2016 ▸, 2019 ▸). In contrast to the most common SLBs, composed of phospholipids (Richter *et al.*, 2006 ▸), the SLBs obtained by spreading L_3_ NPs are potentially more fragile and less resistant to extensive solvent rinsing, such as that required for implementing the solvent contrast variation method. At the same time, NR is among the few techniques that can provide structural information with a few ångstrom resolution on these types of samples. Therefore, the application of substrate assemblies with an MRL is particularly relevant. As well as the SLB mentioned above, other soft and weakly adsorbed/deposited layers, *e.g.* composed of proteins, can profit from a similar application of substrates with MRLs in combination with PNR experiments. Recently, Zubayer *et al.* (2025 ▸) investigated the optimization of MRLs for NR using a model-free comparison of candidate MRL systems and identified CoTi alloys as promising materials. Their work highlights the importance of tuning the nuclear and magnetic scattering length densities of the reference layer in order to improve spin-dependent sensitivity. In the present study, we address a complementary problem: rather than comparing MRL materials at the level of calculated reflectivity response, we optimize a specific soft-matter PNR experiment using a Fisher information (FI) objective that explicitly incorporates counting statistics and parameter correlations.

There is increased interest in employing PNR for studying soft thin films, but experiments are extremely expensive to run and difficult to access – usually requiring a lead time of several months. It is therefore important to make the best possible use of experimental beam time. We extend the exploration of substrate assemblies with MRLs and silicon oxide capping layers for the investigation of soft-matter samples at the solid–liquid interface by theoretical modelling and optimizing the substrate composition and structure. The software *HOGBEN* (Durant *et al.*, 2022*b* ▸) was recently designed for the optimization of experimental setups to ensure that the data extracted from PNR and NR experiments contain as much information as possible. Here we implement this methodology for the optimization of the MRL thickness, nuclear and magnetic SLDs, and the silicon oxide capping layer thickness. In this optimization process, the sample structure is considered known and fixed to that of a reference sample, which we selected to be a GMO-50/DGMO SLB recently investigated by PNR (Luchini *et al.*, 2025 ▸). We show that this optimization provides unique input into the design of a new generation of MRLs that improve information extracted from PNR soft-matter experiments. The correlation between different parameters that determine the reflectivity curve makes it challenging to optimize the setup in any other way than the presented approach. We show that the MRL parameters can be tailored in such a way that the data quality is preserved while measurement time is reduced. Although specifically implemented in the context of an SLB PNR experiment, the presented approach can be easily adapted to other soft-matter systems as well as to other kinds of PNR/NR experiments.

## 2. Theory and modelling

### 2.1. Polarized neutron reflectometry

**Figure 1:**
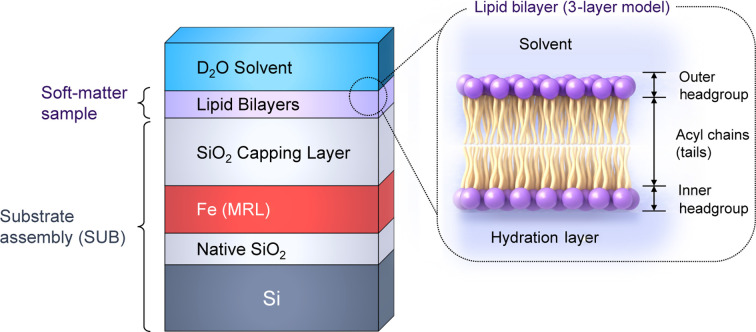
Sketch of the structure studied in the experiment. The substrate assembly consists of Si with native oxide, an Fe MRL and an SiO_2_ capping layer. The soft-matter sample is a lipid bilayer deposited and investigated in D_2_O solvent. The lipid bilayers are modelled as three layers with the acyl chains sandwiched between the inner and outer headgroups. A thin D_2_O hydration layer between the capping layer and the inner lipid headgroup was also part of the model. The bilayer inset is a schematic illustration generated with AI assistance and edited by the authors for clarity.

In this study, we re-analysed experimental data previously collected at the POLREF reflectometer at the ISIS Neutron and Muon Source (Didcot, UK). The reference SLB was obtained by exposing the substrate to a dispersion of L_3_ NPs with composition of 70 wt% GMO-50/DGMO [60/40 (wt/wt)] and 30 wt% polysorbate 80. Experimental details on data collection and sample preparation can be found elsewhere (Luchini *et al.*, 2025 ▸). Analysis of these data provided the sample structure that was later used for the calculations performed within *HOGBEN*. During data analysis, the sample is modelled as a stack of layers, each having a different SLD. It is assumed that the SLD profile varies only in the *z* direction, perpendicular to the sample surface. That is, the off-specular reflectivity is negligible. An overview of the NR/PNR forward model [SLD profile → *R*(*Q*)] and the layer-stack parametrization used here for specular reflectometry is shown by Yakymenko (2025 ▸). Each layer is characterized by thickness, roughness and SLD. Data analysis was performed with the Python package *refnx* (Nelson & Prescott, 2019 ▸; Nelson, 2024 ▸). Fig. 1 ▸ shows a schematic illustration of the layered structure of the substrate assembly and the SLB used in the PNR experiments. The substrate assembly consists of Si with its native oxide, an Fe MRL and a silicon oxide capping layer. As discussed by Luchini *et al.* (2025 ▸), the SLB was described as two outer headgroup layers and an intermediate acyl chain region.

Using *refnx*, PNR data collected for the SUB before lipid deposition were analysed to extract the parameters describing the substrate layers. The SUB parameters were then fixed in the subsequent analysis of the data collected after SLB formation. Co-refinement was performed on data for both neutron spin directions. Results of the data analysis are summarized in the supporting information and are overall in agreement with the previous publication (Luchini *et al.*, 2025 ▸).

### 2.2. *HOGBEN* software

*HOGBEN* is a codebase for sensitivity-guided design of NR experiments and sample architectures using a metric derived from the FI (Durant *et al.*, 2022*a* ▸,*b* ▸, 2021 ▸). In this case, FI quantifies how strongly the expected reflectivity is sensitive to changes in a chosen set of model parameters and provides a lower bound on the achievable parameter covariance (Cramér–Rao bound). In practice, larger FI implies that the parameters of interest can be constrained more tightly by a dataset acquired under the corresponding experimental conditions.

For a reflectometry model evaluated at points *Q*_*i*_ with predicted reflectivities *r*_*i*_ = *R*(*Q*_*i*_; **θ**), where **θ** denotes the vector of model parameters of interest to be inferred from the data, *HOGBEN* follows Durant *et al.* (2021 ▸, 2022*b* ▸) and constructs the FI matrix 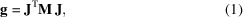
where *J*_*ij*_ = ∂*r*_*i*_/∂θ_*j*_ is the Jacobian and **M** is a diagonal weighting matrix determined by Poisson counting statistics. In the formulation (Durant *et al.*, 2021 ▸), *M*_*ii*_ = *s*_*i*_/*r*_*i*_, where *s*_*i*_ is the incident neutron counts in bin *i*. This makes FI effectively a sensitivity analysis (∂*r*/∂θ) weighted by counting statistics: a datapoint is informative when small parameter changes produce large changes in *r*_*i*_ and when the measurement is supported by high neutron counts.

Importantly, because *s*_*i*_ scales linearly with beam time (for fixed flux and binning), the FI matrix scales approximately linearly with the allocated measurement time. Consequently, the Cramér–Rao bound implies that achievable parameter variances scale approximately inversely with time. This provides a use of FI beyond improving constraints: if a target level of parameter precision is considered sufficient (*e.g.* informed by a previous similar experiment), then an FI-guided optimization can be used to reduce measurement time while keeping that target precision by reallocating time/angles/solvents and/or tailoring the sample design (Durant *et al.*, 2022*b* ▸).

Because FI depends on the parametrization and units, Durant *et al.* (2022*b* ▸) introduce a bounds-based ‘importance scaling’ to place parameters with different units on a common dimensionless scale. In our implementation, we specify physically meaningful bounds for each parameter included in the FI calculation, and *HOGBEN* uses these bounds when evaluating the eigenvalue-based design objective (Durant *et al.*, 2022*a* ▸).

We summarize experiment quality with a single scalar information metric (arbitrary units), 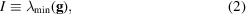
where 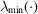 denotes the minimum eigenvalue. We adopt this maximin objective because correlations between parameters can leave a poorly constrained linear combination of parameters even when individual sensitivities appear favourable: the smallest-eigenvalue mode corresponds to the direction that is least informative in parameter space; therefore, maximizing *I* improves the weakest attainable constraint (Durant *et al.*, 2022*b* ▸).

For clarity, when comparing a baseline design, *i.e.* the initial model as depicted in Fig. 1 ▸ and based on the structure in the reference PNR experiment (Luchini *et al.*, 2025 ▸), with an optimized design we report an information gain, *G* (FI gain factor),
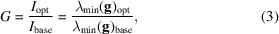
*i.e.* the ratio of *I*_opt_ and *I*_base_, the information metrics after and before optimization, respectively. Values of *G* > 1 indicate that the optimized configuration yields higher information content in this maximin sense. Equivalently, for fixed flux and model assumptions, the same target precision can, in principle, be reached in reduced beam time by approximately a factor of *G*. We emphasize that all gain factors *G* reported below are FI-based predictions for the chosen parametrization and bounds, not experimentally measured reductions in beam time. Validation requires synthesizing the optimized substrate assemblies and comparing fitted parameter uncertainties at matched beam time.

The computational cost of evaluating the information metric is low, enabling rapid exploration of experimental conditions and sample-design variables (Durant *et al.*, 2021 ▸). In this work, the components of **θ** were the SLB parameters, while the MRL and the silicon oxide capping layer were treated as design variables. Further derivation and background on FI for NR/PNR are provided by Yakymenko (2025 ▸). The optimization workflow implemented in a *Jupyter* notebook and used to generate the optimization results and gain factors reported here is archived on Zenodo (Yakymenko & Cooper, 2026 ▸).

## 3. Results and discussion

We used the *HOGBEN* software to optimize key structural features of the MRL recently used to investigate SLBs. We considered two cases: (I) optimization of the thicknesses of the Fe MRL and the silicon oxide capping layer, and (II) optimization of the SLD (both magnetic and nuclear) of the MRL along with the thicknesses for the Fe MRL and the silicon oxide capping layer. The sensitivity of the PNR data to these parameters is quantitatively expressed by the information metric, which is calculated from the corresponding Fisher matrix as described in Section 2.2. We recall that 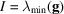 is the smallest eigenvalue of the Fisher matrix and provides a lower bound on the worst-constrained linear combination of SLB parameters. Larger *I* corresponds to lower predicted parameter uncertainties, so maxima in the plotted information metric curves below identify the most informative configurations within the considered ranges. During these calculations the soft-matter sample (*i.e.* SLB) structure was kept fixed to the parameters determined during the data analysis described in Section 2.1 and reported in the supporting information.

**Figure 2:**
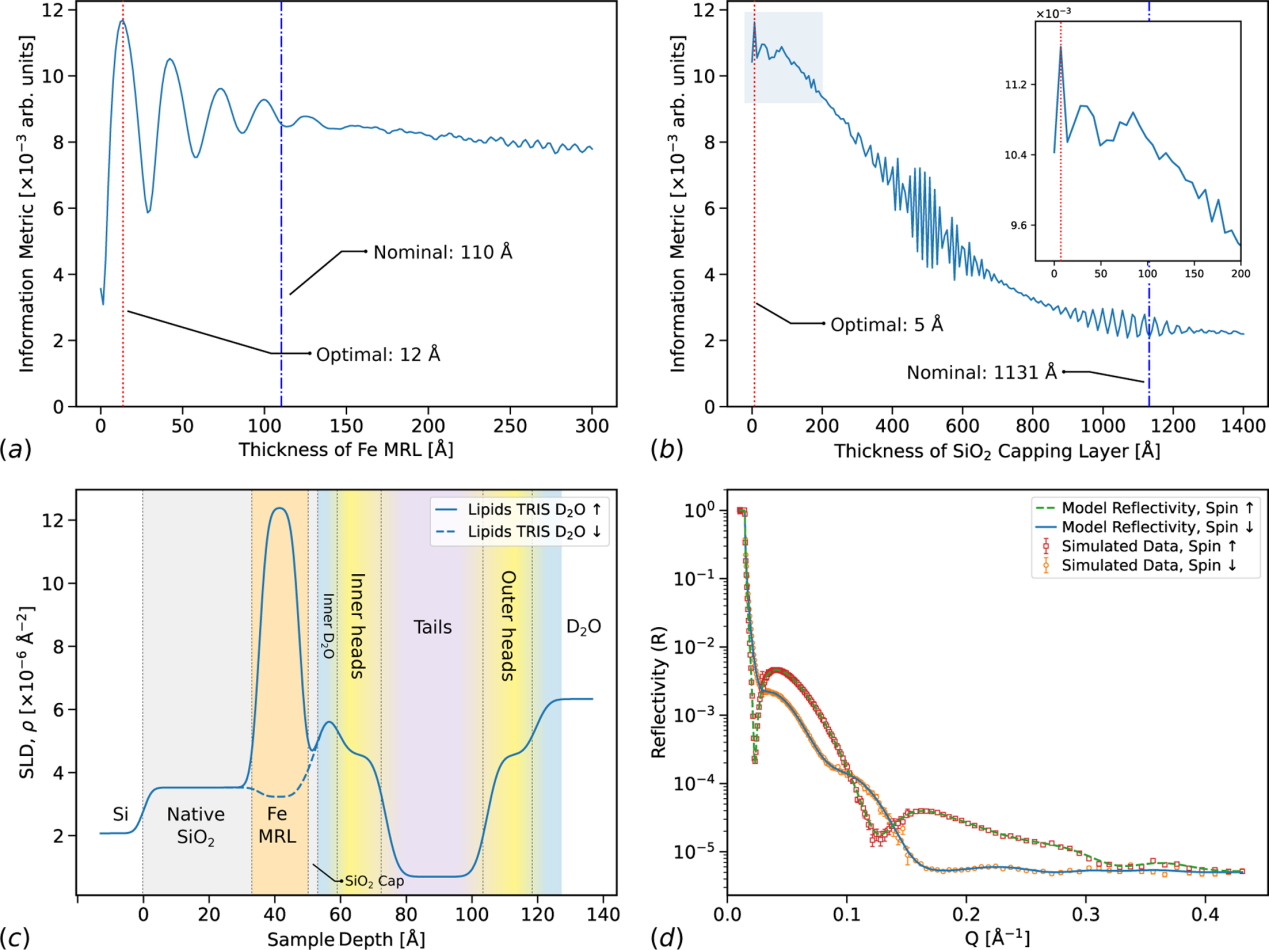
The information metric 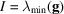 is the minimum eigenvalue of the FI matrix **g** defined in Section 2.2, where larger values correspond to tighter Cramér–Rao lower bounds on the SLB parameters. Plots of *I* as a function of (*a*) Fe MRL thickness and (*b*) SiO_2_ capping layer thickness; (*c*) the resulting SLD depth profile after optimization, and (*d*) the model reflectivity and simulated data for spin-up and -down. The Fe thickness decreased from the nominal value of 110.3 Å to the optimal value of 12.3 Å. The SiO_2_ thickness was significantly reduced from 1131.3 Å down to the optimal value of 5.49 Å.

For case (I), Figs. 2 ▸(*a*) and 2 ▸(*b*) show how the information metric depends on the thickness of the Fe MRL over the range [0, 300] Å and the thickness of the silicon oxide capping layer over the range [0, 1400] Å, respectively. The optimal thicknesses within these bounds were found to be 12.3 and 5.49 Å, respectively, corresponding to a predicted information gain of *G* ≈ 4.5 relative to the reference PNR experiment. For the Fe MRL, the information metric is highly sensitive to the thickness only in the thin-layer regime, where pronounced oscillations are observed; beyond ∼100 Å, the dependence becomes much weaker and the metric remains nearly constant, with a slight downward trend. In contrast, the information metric for the SiO_2_ capping layer shows a clear overall decrease with increasing cap thickness, although this trend is modulated by oscillatory structure. Fig. 2 ▸(*b*) therefore indicates that thinner SiO_2_ capping layers are predicted to be generally more favourable, either reducing the measurement time required to achieve a given precision or improving parameter uncertainties at fixed measurement time. The regions of more pronounced oscillations in the information metric that can be seen for both Fe and SiO_2_ arise from interference effects in the multilayer stack, which cause the sensitivity of the reflectivity to structural parameters to vary non-monotonically with layer thickness.

The unconstrained FI optimum favours very thin SiO_2_ caps (below ∼10 Å), but such values are not realistic for soft-matter PNR: very thin oxide caps are less robust in the aqueous and saline environments typical of these experiments, and accordingly thicker SiO_2_ coatings have been developed to improve the stability of Fe-based MRLs (Dikaia *et al.*, 2024 ▸); the cap in the reference PNR experiment was ∼1000 Å (Luchini *et al.*, 2025 ▸). Experimentally screening the full intermediate range of cap thicknesses would be costly. Instead, we use the fast FI calculation to identify a practical cap-thickness window of 200–400 Å, well below the reference value but thick enough to retain the protective function of the cap, within which the predicted information gain remains substantial (*G* ≈ 2.7, evaluated at the reference Fe MRL thickness of 110 Å). This 200–400 Å window is the main practical recommendation of the present study, whereas the unconstrained optima reported below should be interpreted as theoretical reference points rather than directly fabricable specifications.

**Figure 3:**
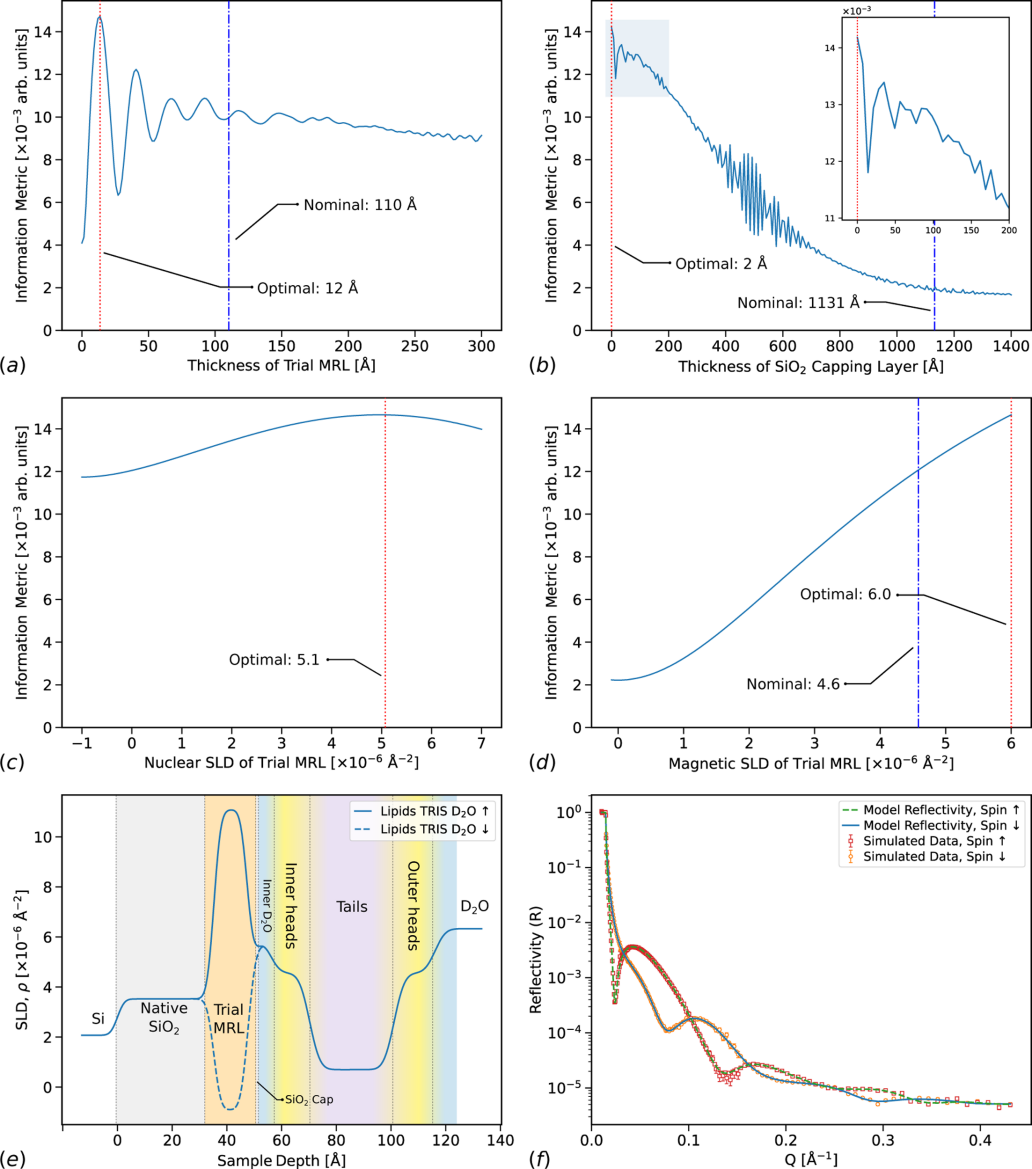
As in Fig. 2 ▸, the information metric 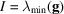 quantifies the FI-predicted constraint on the SLB parameters where higher values are more informative. Plots of *I* as a function of (*a*) trial MRL thickness and (*b*) SiO_2_ capping layer thickness; (*c*) nuclear and (*d*) magnetic SLD of the trial MRL; (*e*) the resulting SLD profile after optimization; and (*f*) corresponding model reflectivity and simulated data for spin-up and -down. The optimal nuclear SLD of the trial MRL reduced to 5.1 × 10^−6^ Å^−2^ from a nominal value of 7.8 × 10^−6^ Å^−2^ for Fe. On the other hand, the optimal magnetic SLD of the trial MRL reached 6.0 × 10^−6^ Å^−2^, the upper bound of the varying parameter range, in contrast to 4.6 × 10^−6^ Å^−2^ for Fe from the measurements performed with POLREF.

For case (II), the thickness of the trial MRL and the silicon oxide capping layer was varied over the intervals [0, 300] Å and [0, 1400] Å, respectively, and the nuclear and magnetic SLDs were varied over the intervals [−1, 7] × 10^−6^ Å^−2^ and [−0.1, 6] × 10^−6^ Å^−2^, respectively. The resulting optimal values of the thicknesses of the trial MRL and silicon oxide layer were 12.2 and 2.1 Å, respectively (Fig. 3 ▸). These are very close to the values found for the Fe MRL in case (I), which suggests that the optimal silicon oxide layer thickness is largely insensitive to the choice of material for the MRL. The optimal value for the trial MRL nuclear SLD was 5.1 × 10^−6^ Å^−2^ and for the magnetic SLD was 6.0 × 10^−6^ Å^−2^.

Our calculations, summarized in Table 1 ▸, indicate that an MRL with a lower nuclear SLD and higher magnetic SLD than Fe is predicted to further improve the outcome of PNR experiments, either by reducing the measurement time required to reach a given uncertainty or by reducing parameter uncertainties at fixed beam time. Relative to the fitted Fe values in Table S1 of the supporting information (nuclear SLD = 7.82 × 10^−6^ Å^−2^, magnetic SLD = 4.58 × 10^−6^ Å^−2^), the FI optimum therefore points toward a magnetic layer with smaller nuclear contrast and enhanced magnetic contrast. A plausible candidate family for future studies is Fe–Co alloys, since Co has a substantially lower nuclear SLD than Fe, of the order of 2.3 × 10^−6^ Å^−2^, while Fe−Co alloys are known to sustain magnetizations higher than that of pure Fe (López-Martín *et al.*, 2024 ▸). In this sense, the present FI results do motivate a materials design search within the Fe–Co system as a realistic starting point for future experimental optimization. This trend is broadly consistent with the recent identification of CoTi alloys as promising MRL candidates in reflectivity-based sensitivity comparisons (Zubayer *et al.*, 2025 ▸). However, the comparison should not be taken as quantitative, since the present optimization was carried out for a capped substrate assembly tailored to soft-matter samples, whereas Zubayer *et al.* (2025 ▸) considered a different layer architecture.

## 4. Conclusions

We have discussed the design of substrate assemblies with an Fe-based MRL and a silicon oxide capping layer, in order to expand their application in PNR experiments on soft-matter samples. We performed quantitative calculations using the *HOGBEN* software to assess the thicknesses of the Fe MRL and the silicon oxide capping layer that provide the highest sensitivity of PNR data to determine the structure of an SLB. The main input for future substrate-assembly design was to considerably reduce the thickness of the silicon oxide capping layer to 200–400 Å, which is sufficient to protect the MRL from solvent. Within this practical window, and at the reference Fe MRL thickness, the FI analysis predicts an information gain of *G* ≈ 2.7 – a substantial improvement that is achievable without departing from current substrate-fabrication practice.

In our calculation, we also evaluated the scenario of replacing the MRL with a material other than Fe by allowing both the nuclear and the magnetic SLDs of the MRL to be optimized along with its thickness and the thickness of the silicon oxide layer. We demonstrate that an MRL material with a lower nuclear SLD and a larger magnetic SLD compared with Fe would be ideal.

Our approach will help design PNR experiments to deliver the highest sensitivity of the determined parameter for a particular system. It also allows us to propose new layered structures containing an MRL with properties favourable for experiments. The key result is that the benefits of the MRL depend strongly on the capping layer thickness. We emphasize that the gain factors reported here are FI-based predictions and that experimental validation on substrate assemblies fabricated according to these recommendations remains future work.

## Supplementary Material

The characterization of the substrate assembly in three solvents and the subsequent lipid bilayer characterization with PNR. DOI: 10.1107/S160057672600539X/po5177sup1.pdf

## Figures and Tables

**Table 1:** Results after employing the *HOGBEN* package to optimize the layers in the SUB with regards to the SLB parameters Case (I): Fe MRL and SiO_2_ cap thicknesses were optimized. Case (II): the thickness and nuclear and magnetic SLDs of a new (trial) MRL as well as the thickness of the SiO_2_ cap were optimized. The rightmost column shows the information gain factor *G* defined in Section 2.2. Starting with the fitted model from the reference PNR experiment conducted at ISIS, *HOGBEN* optimization returned the configuration denoted by ‘opt.’. For case (II), nSLD and mSLD denote nuclear and magnetic SLD, respectively.

	Range (Å)	Opt. (Å)	SLD range (× 10^−6^ Å^−2^)	Opt. SLD (× 10^−6^ Å^−2^)	Gain *G*
Case (I)					
Fe	0–300	12.3	–	–	4.5
SiO_2_	0–1400	5.5	–	–	4.5

Case (II)					
Trial MRL	0–300	12.21	nSLD: [−1, 7]	5.1	5.6
			mSLD: [−0.1, 6]	6.0	5.6
SiO_2_	0–1400	2.1	–	–	5.6

## Data Availability

The PNR data supporting this study are available from the ISIS Neutron and Muon Source data repository at https://doi.org/10.5286/ISIS.E.RB2220651-1 (Nylander *et al.*, 2023 ▸). *Jupyter* notebooks with data fitting and optimization are archived at Zenodo (https://doi.org/10.5281/zenodo.19323498) (Yakymenko & Cooper, 2026 ▸) and developed at https://github.com/ivaya443/HOGBEN-optimising-PNR.
