# Supplementary material for: Improving polarized neutron reflectometry experiments on soft-matter samples: optimization of the solid substrate structure
Source: J Appl Crystallogr. 2026 Jul 22;59(Pt 4):1192–9. doi: 10.1107/S160057672600539X (PMC13440064; doi:10.1107/S160057672600539X)
Supplement: Supplementary file 1 [file j-59-01192-sup1.pdf]

JOURNAL OF
APPLIED
CRYSTALLOGRAPHY

Volume 59 (2026)

Supporting information for article:

Improving polarized neutron reflectometry experiments on soft-matter samples: optimization of the solid substrate structure

Ivan P. Yakimenko, Alessandra Luchini, Joshaniel F. K. Cooper, Sebastian Köhler, Tommy Nylander, Sean Langridge, Christy J. Kinane, Andrew J. Caruana, Björgvin Hjörvarsson, Max Wolff, Alexei Vorobiev, Sergiy Valyukh, Fredrik Eriksson, Jens Birch and Kenneth Järrendahl

Improving polarised neutron reflectometry experiments on soft-matter samples: optimisation of the solid substrate structure – Supplementary Material

Ivan P. Yakimenko [a], Alessandra Luchini [b,c], Joshaniel F. K. Cooper [d], Sebastian Köhler [e,f], Tommy Nylander [e,f,g,h], Sean Langridge [i], Christy J. Kinane [i], Andrew J. Caruana [i], Björgvin Hjörvarsson [j], Max Wolff [j], Alexei Vorobiev [j,k], Sergiy Valyukh [a], Fredrik Eriksson [a], Jens Birch [a], and Kenneth Järrendahl [a]

[a]Thin Film Physics Division, Department of Physics, Chemistry and Biology (IFM), Linköping University, SE-581 83 Linköping, Sweden
[b]Department of Physics and Geology, University of Perugia, via Alessandro Pascoli, 06123 Perugia, Italy
[c]Department of Physics and Geology, CNR-IOM c/o University of Perugia, via Alessandro Pascoli, 06123 Perugia, Italy
[d]European Spallation Source ERIC, Lund, Sweden
[e]Division of Physical Chemistry, Department of Chemistry, Lund University, 221 00 Lund, Sweden
[f]LINXS Institute of Advanced Neutron and X-ray Science, 223 70 Lund, Sweden
[g]NanoLund, Lund University, Lund, Sweden
[h]School of Chemical Engineering and Translational Nanobioscience Research Center, Sungkyunkwan University, 16419 Suwon, Republic of Korea
[i]ISIS Neutron and Muon Source, Rutherford Appleton Laboratory, Chilton, Oxon OX11 0QX, United Kingdom
[j]Department of Physics and Astronomy, Material Physics, Uppsala University, SE-751 20 Uppsala, Sweden
[k]Institut Laue-Langevin, 71 Avenue des Martyrs, CS 20156, 38042 Grenoble Cedex 9, France

1 Details of layers deposition

Si-blocks of 80×50 mm^2 with a total thickness of 14.5 mm were used with their thin native SiO_2 for the the deposition. An Fe layer of 110 Å was grown followed by 1100 Å SiO_2. The substrate assemblies (SUBs) were purchased from Königssystems (Königssystems UG, 2025) and were also used in our other study (Luchini *et al.*, 2025).

The SUBs used for this study were mounted in a solid–liquid cell connected to a high-performance liquid chromatography pump for solvent injection. The temperature of the cells was kept at 25 °C during the measurements. PNR measurements were performed in contact with three different solvents: water H_2O, heavy water D_2O, or silicon matched water (SMW) to determine the structural parameters of the SUBs without lipid bilayers. The lipid suspensions with compositions detailed in the main text were prepared as reported in (Luchini *et al.*, 2025). The cell was rinsed with a tris(hydroxymethyl)aminomethane (TRIS) buffer and upon formation of the supported lipid

bilayers, PNR data were collected in the D_2O solvent.

2 Details of NR POLREF experiment

The time-of-flight neutron reflectometer POLREF at the ISIS Neutron and Muon Source in Chilton, UK uses a polarised wavelength band of 2 − 14 Å with polarisation efficiency of 98% at 2 Å and 99.5% or more above 5 Å. Polarised neutrons with different spin direction were reflected from the solid-liquid interface before being detected. Data sets consisted of reflectivity for neutron beam polarisation parallel and antiparallel to the magnetisation of the Fe layer (Nylander *et al.*, 2023). The reflectivity data sets were obtained at room temperature in an external magnetic field of H = 100 Oe generated by a set of permanent magnets.

3 Details of neutron reflectometry measurements and analysis

3.1 Characterising the substrate assembly in various solvents

Figure S1 shows the reflectivity curves for the SUB measured in D_2O, H_2O, and SMW. Reflectivity data for both spin-up and spin-down polarised neutron beams are shown together with the corresponding fits. For clarity, the experimental data (squares/circles for spin-up/spin-down) and fitted curves (dashed/solid for spin-up/spin-down) for H_2O and SMW are offset by factors of 10^3 and 10^6, respectively. The SLD profiles obtained from the fits are shown in Fig. S2. For all three solvents, the nuclear SLD profiles of the non-magnetic layers are identical within the fitted model. In contrast, the SLD profile separates into spin-up and spin-down branches within the Fe layer in Fig. S2, confirming that the Fe layer is magnetised and provides magnetic contrast. The resulting SLD values and layer thicknesses are summarised in Table S1.

Figure S3 shows the corner plots produced by *refnx* using Bayesian MCMC sampling, illustrating the posterior distributions of the fitted parameters. These plots provide information on parameter uncertainties and correlations between parameters in the SUB model. For two normally distributed, uncorrelated parameters, the corresponding two-dimensional posterior is circular or nearly isotropic, whereas correlated parameters produce elongated distributions. Overall, most pairwise distributions are close to isotropic, indicating weak parameter correlations. The one-dimensional posterior distributions shown along the main diagonal of Fig. S3 are generally well defined and approximately Gaussian, with the exception of the Fe roughness parameter. Parameter uncertainties were derived from these posterior distributions and are reported in Table S1. Because scale, background, and resolution were fixed during the analysis, their contribution to the total uncertainty is not propagated into the reported credible intervals.

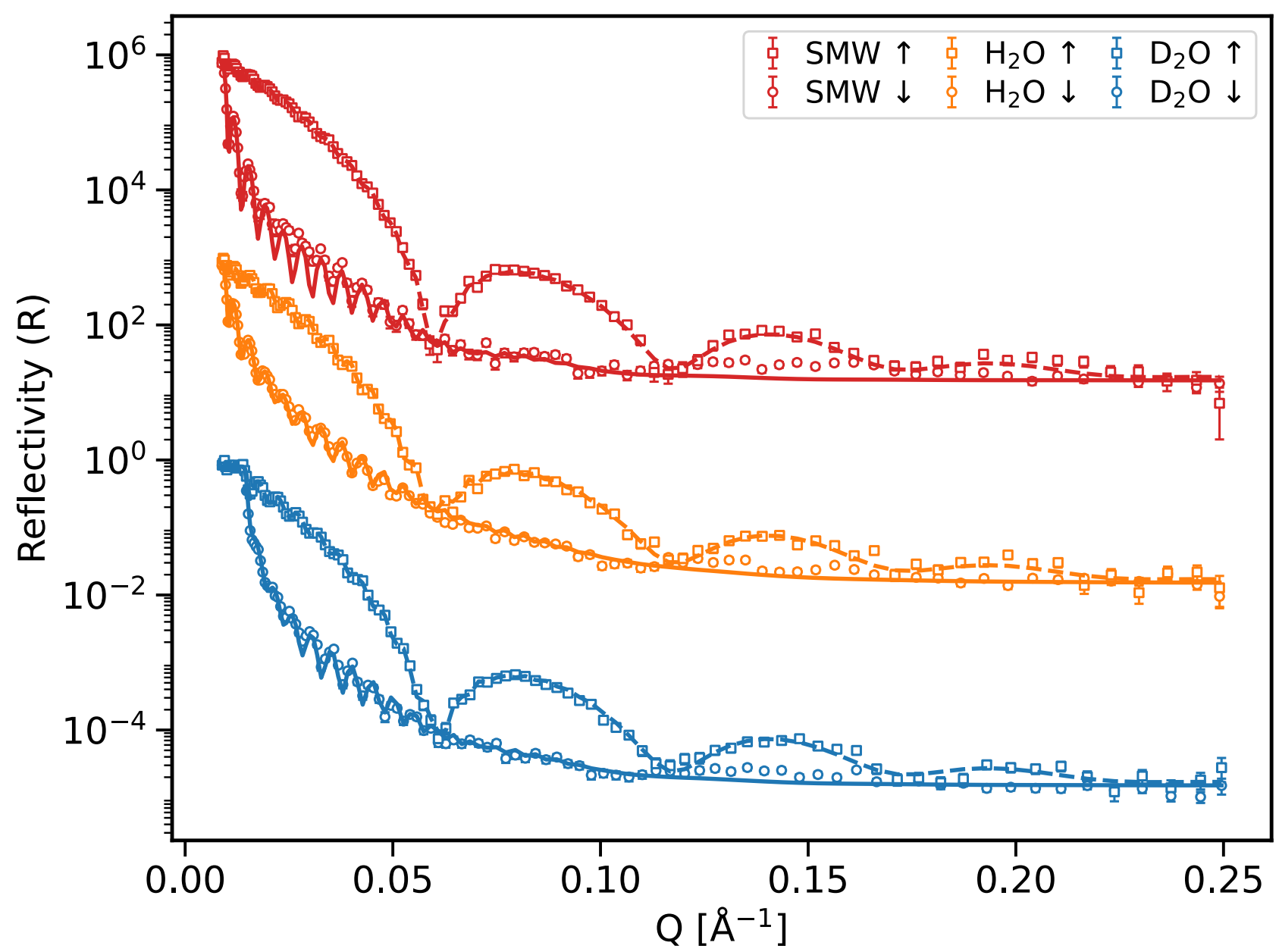

Figure S1: Experimental (squares/circles – spin up/down) and simulated (dashed/solid – spin up/down) reflectivity data for the substrate assembly in three different solvents (D_2O, H_2O and SMW). Reflectivity data for H_2O and SMW are offset by 10^3 and 10^6 for clarity.

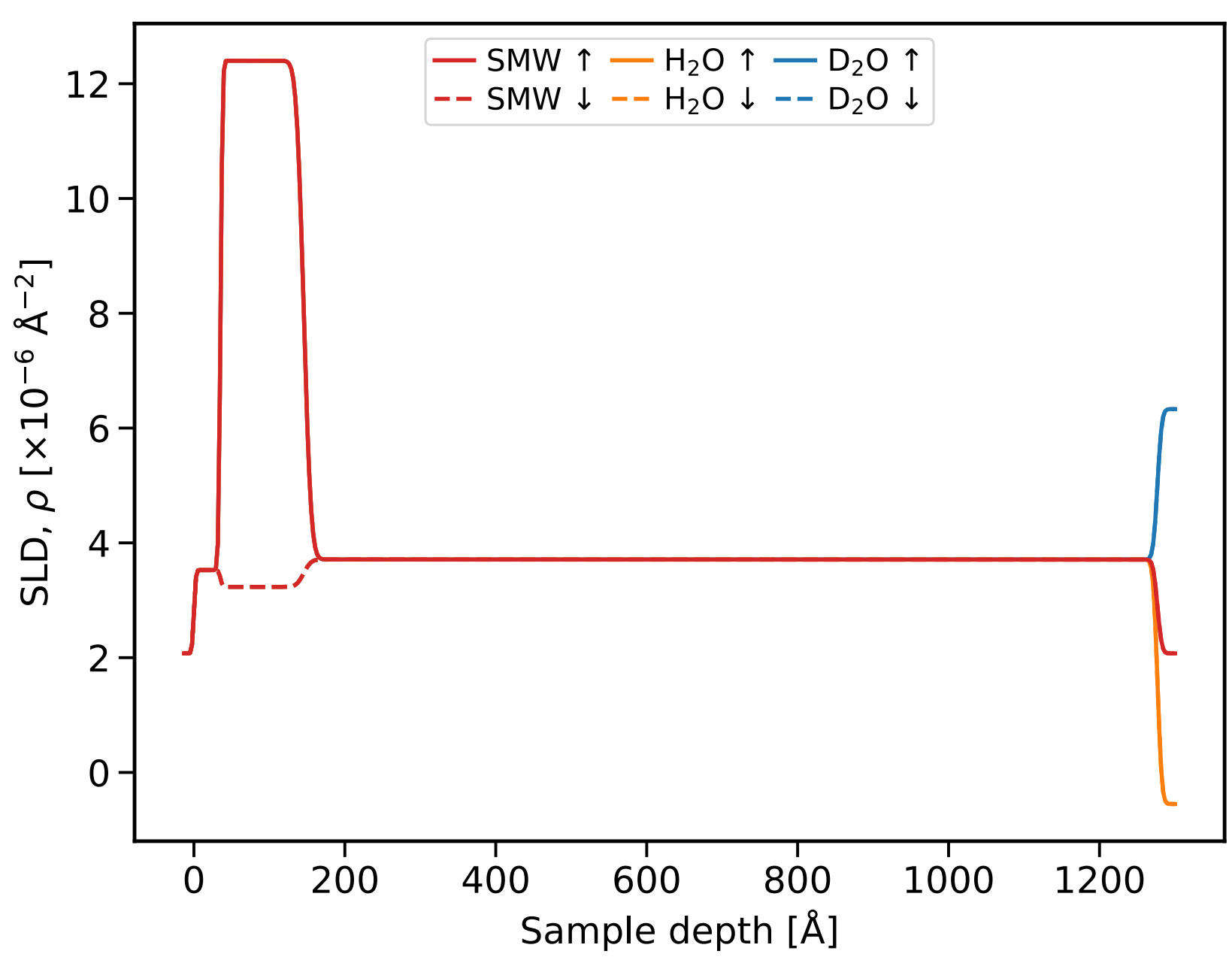

Figure S2: The obtained SLD profiles for the substrate assembly in three different solvents. Due to the co-refinement all curves are on top of each other except for the Fe layer (separating curves for spin-up and spin-down cases), and in the solvent region (separating curves for D_2O, H_2O and SMW).

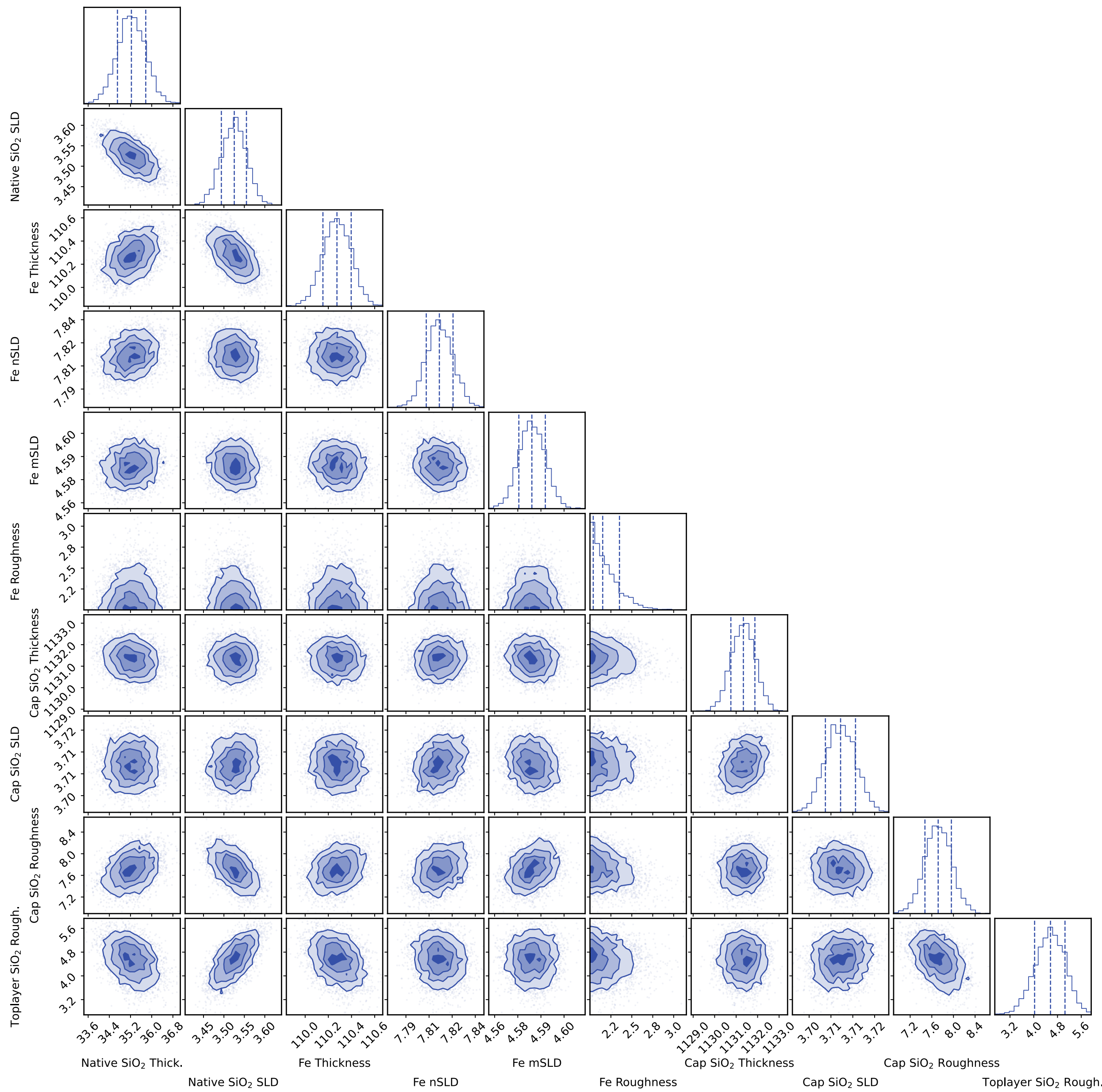

Figure S3: The resulting corner plot after characterising the SUB.

Table S1: The first stage – characterisation of the SUB. Fitted values of layer thickness, roughness, and SLD are reported for the different layers. Uncertainties correspond to posterior credible intervals obtained from Bayesian MCMC sampling within the adopted reflectometry model. These intervals are conditional on the chosen model and on fixed instrumental terms such as scale, background, and resolution, and therefore primarily reflect internal parameter uncertainty within that framework. Additional uncertainty associated with model choice, parametrisation, or fixed experimental terms is not included and may increase the effective uncertainty in practice.

Thickness [Å]	
Native SiO_2	35.2 ± 0.5
Fe	110.3 ± 0.1
SiO_2 cap	1131.3 ± 0.6
**Roughness [Å]**	
Native SiO_2 / Fe	2.2 ± 0.2
Fe / SiO_2 cap	7.7 ± 0.2
SiO_2 cap / Solvent	4.6 ± 0.5
**SLD [$\times 10^{-6}$ Å^{-2}]**	
Native SiO_2	3.53 ± 0.03
Fe (Nuclear)	7.82 ± 0.01
Fe (Magnetic)	4.58 ± 0.01
SiO_2 cap	3.710 ± 0.003

3.2 Characterising the lipid bilayers

The next series of experiments were performed with deposited lipid bilayers in D_2O solvent after rinsing with TRIS buffer. Experimental and simulated reflectivity data are shown in Fig. S4. In Figs. S5 and S6 the SLD profiles and corner plots, respectively, are presented. The structural parameters obtained from the data analysis are reported in Table S2. The values of uncertainty for parameters can be extracted from the width of Gaussian distributions in Fig. S6.

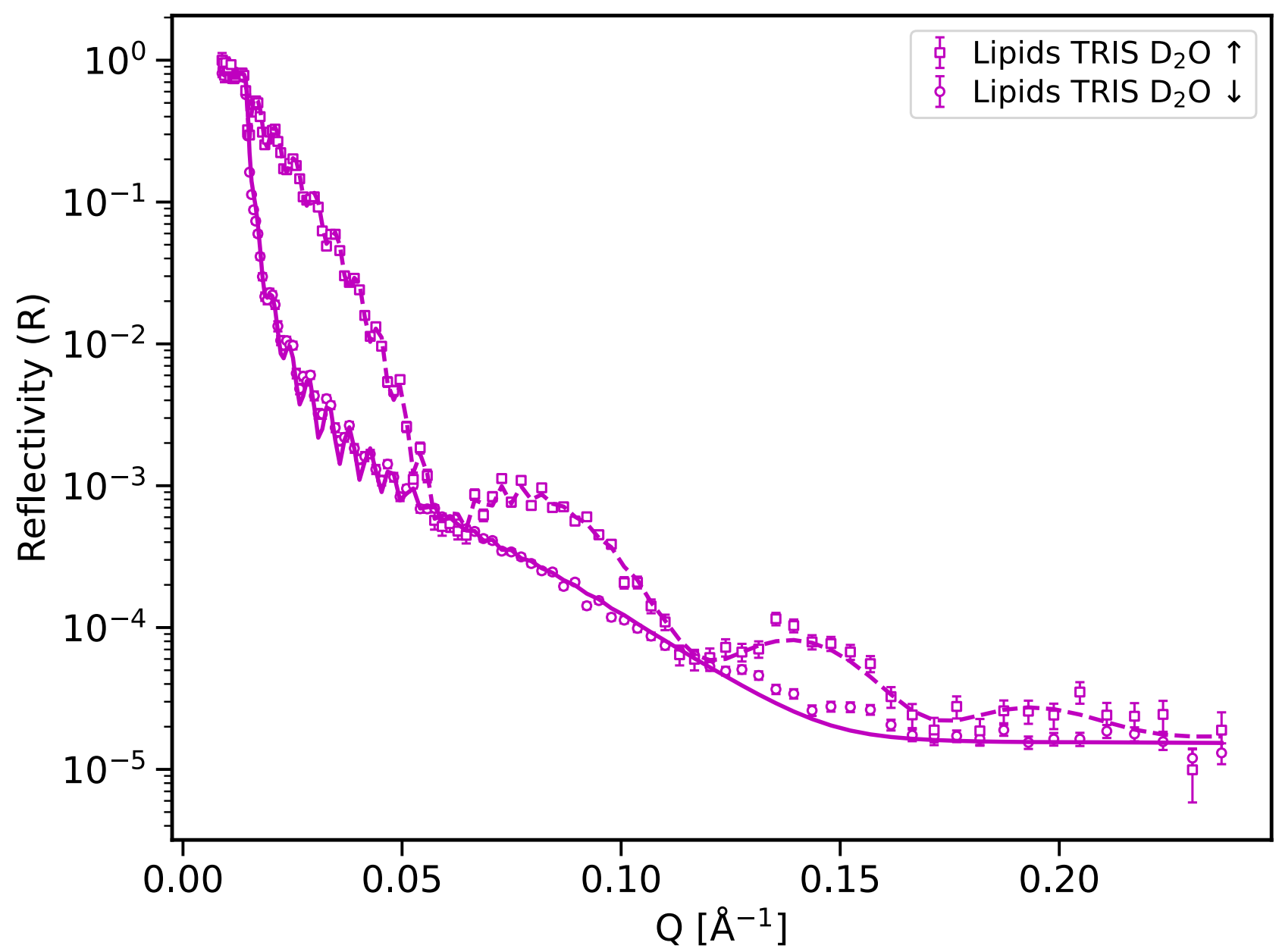

Figure S4: Experimental (squares/circles – spin up/down) and simulated (dashed/solid – spin up/down) reflectivity data for substrate assembly with deposited lipid bilayers on top in D_2O after rinsing with TRIS buffer.

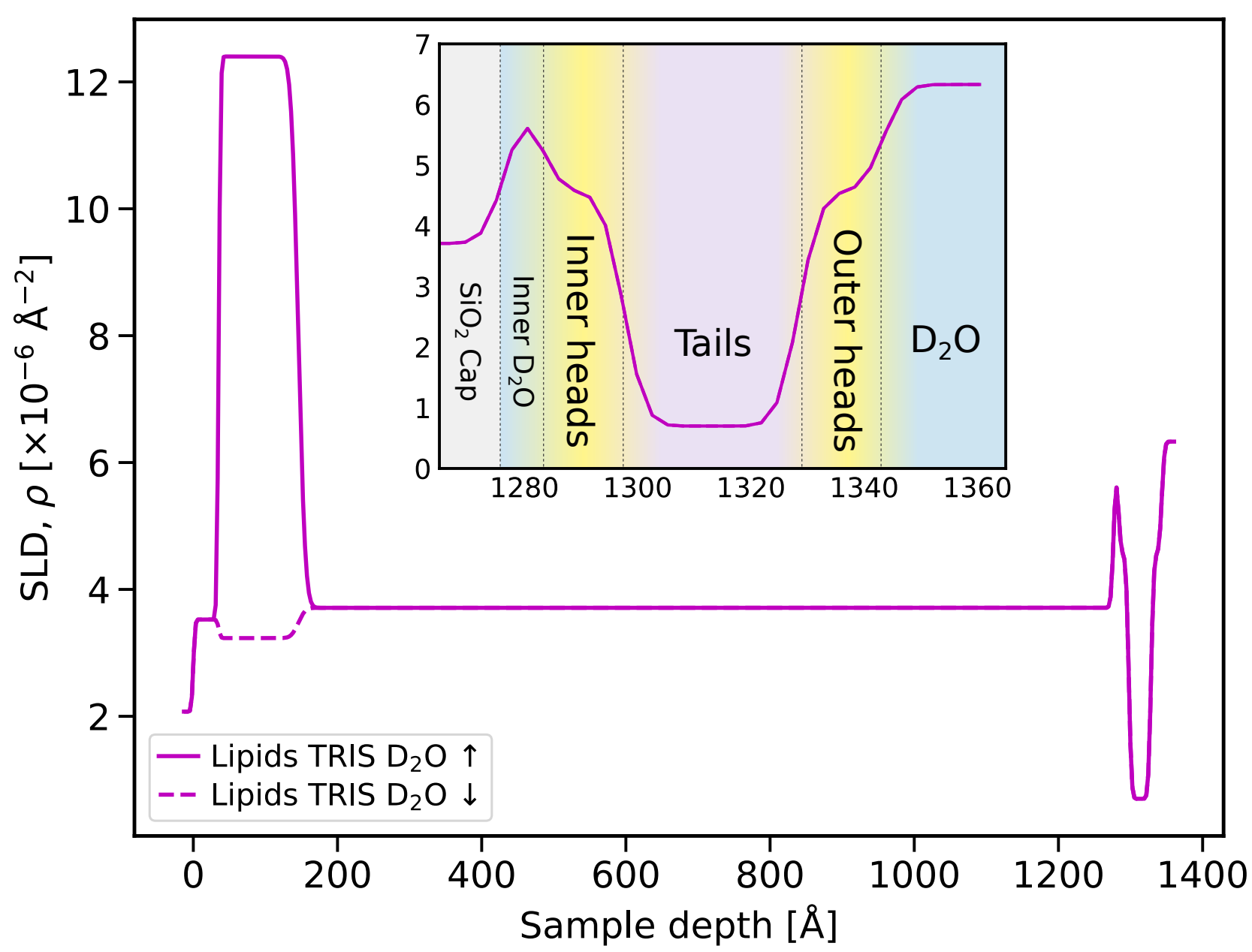

Figure S5: SLD profile with lipid bilayers deposited onto the SUB in D_2O. The lipid bilayers are split into three layers – inner headgroup, tails and outer headgroup. While modelling, a thin hydration layer of D_2O was situated between the capping layer and the inner lipid headgroup layer.

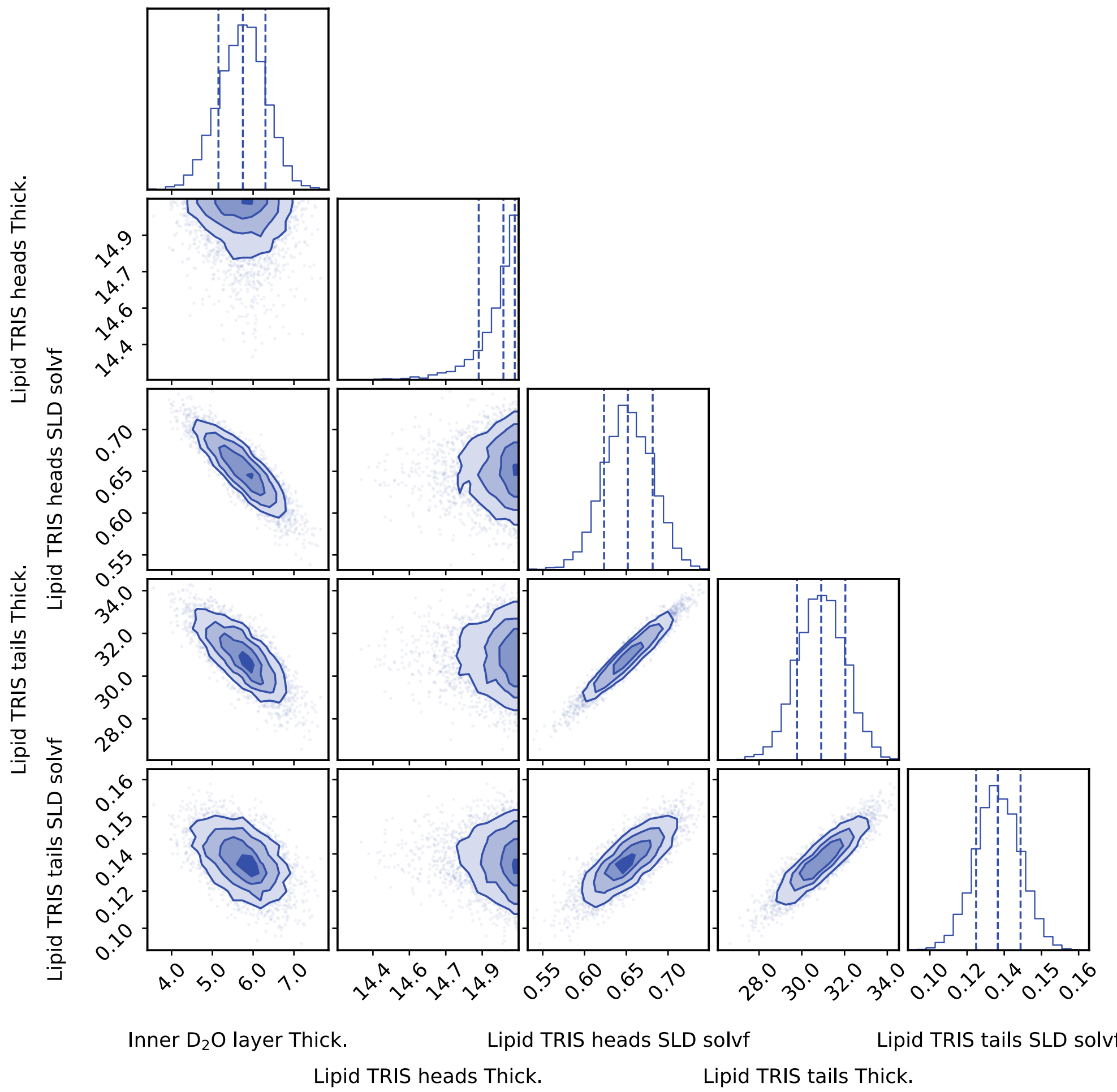

Figure S6: Corner plot for SUB with lipid bilayers deposited on top in D_2O after rinsing with TRIS buffer.

Table S2: The second stage – characterisation of the deposited lipid bilayers after rinsing with TRIS buffer, with the substrate assembly parameters fixed. Fitted values of the thicknesses and SLDs are reported for the three-layer bilayer model comprising the inner headgroup, tail, and outer headgroup regions. In these fits, the substrate assembly parameters were fixed to the values obtained in Table S1.

Thickness [Å]	
D_2O hydration layer between cap / lipids	5.7 ± 0.6
Lipid headgroups (inner/outer)	14.9 ± 0.1
Lipid tails	30.9 ± 1.1
Solvent volume fraction, ϕ_s	
Lipid headgroups (inner/outer)	0.65 ± 0.03
Lipid tails	0.13 ± 0.01

References

Königssystems UG (2025). Home | Koenigssystems. Accessed on August 28, 2025. https://www.koenigssystems.de

Luchini, A., Machingauta, M. R., Köhler, S., Gilbert, J., Yakimenko, I. P., Birch, J., Järrendahl, K., Cooper, J. F., Stendahl, S., Langridge, S., Kinane, C., Caruana, A. J., Dikaia, O., Goikhman, A., Vorobiev, A., Devishvili, A., Hjörvarsson, B. & Nylander, T. (2025). *Journal of Colloid and Interface Science*, **697**, 137879.

Nylander, T., Hjörvarsson, B., Köhler, S., Langridge, S., Caruana, A. J., Järrendahl, K., Birch, J., Yakymenko, I., Wolff, M., Vorobiev, A., Luchini, A. & Cooper, J. F. (2023). Using magnetic reference layers to unravel the interaction of proteins with complex lipid bilayers.
